# Supplementary material for: Age Differences in Attitudes towards Older People in Poland
Source: Int J Environ Res Public Health. 2021 Jun 26;18(13):6869. doi: 10.3390/ijerph18136869 (PMC8297323; doi:10.3390/ijerph18136869)
Supplement: Supplementary file 1 [file ijerph-18-06869-s001.zip › ijerph-1242717-supplementary.pdf]

Table S2a. Results of the Satisfaction with Life Scale (SWLS) of young people and seniors.

Statement	Group	Points—answer/number of people						
		7	6	5	4	3	2	1
		Strongly agree	Agree	Slightly agree	Neither agree nor disagree	Slightly disagree	Disagree	Strongly disagree
In most ways my life is close to my ideal	I	12	29	58	38	22	14	4
	II	2	13	55	39	40	26	2
The conditions of my life are excellent	I	20	54	67	17	9	6	4
	II	6	42	52	34	32	10	1
I am satisfied with my life	I	14	60	59	22	6	10	6
	II	7	48	54	40	21	7	0
So far I have gotten the important things I want in life	I	8	55	43	36	15	10	10
	II	4	55	62	27	17	10	2
If I could live my life over, I would change almost nothing	I	16	21	43	15	49	20	13
	II	7	23	30	21	57	34	5
Average degree of satisfaction with life					Group I	23.2 ± 6.5 points		
					Group II	21.8 ± 5.1 points		
Minimum points					Group I	5		
					Group II	10		
Maximum points					Group I	35		
					Group II	34		
Calculated sten value					Group I	6.6 ± 2.3 sten		
					Group II	5.96 ± 1.8		
Minimum sten					Group I	1		
					Group II	2		
Maximum sten					Group I	10		
					Group II	10		

Table S3a. Results on the Self-Assessment Scale of Own Person.

Item	Points—reply/group/number of people									
	4 points		3 points		2 points		1 point		0 points	
	Very often		Often		Sometimes		Rarely		Never	
	I	II	I	II	I	II	I	II	I	II
I wish my friends would continue to support me	77	53	60	111	28	11	8	2	4	0
I still feel anxious even if I have acted appropriately	44	36	53	88	58	50	22	3	0	0
I am worried about my future	48	39	44	90	57	44	24	4	4	0
Most of my friends hate me	4	13	11	22	37	83	89	59	36	0
I have less initiative than others	14	21	41	79	63	72	49	5	10	0
I'm nervous about my mental state	26	18	29	76	64	62	42	21	16	0
I'm afraid to look like a fool	56	21	46	79	47	49	24	28	4	0
Others look so much better than me	38	29	41	76	64	60	34	12	0	0
I'm afraid to speak in public when I have something to say	50	57	48	55	51	46	26	19	2	0
I often make mistakes	17	20	42	113	98	44	20	0	0	0
I can't speak nicely or correctly	16	18	38	63	54	65	55	31	14	0
I don't believe in my own strength	29	17	41	72	53	80	46	8	8	0
I don't want my actions to be better than the actions of others	4	7	7	61	49	81	79	28	38	0
I'm too modest	10	59	44	74	77	35	34	9	12	0
I have no purpose in life	9	18	24	41	45	84	53	34	46	0

Many people have a bad opinion of me	8	10	15	45	50	101	90	21	14	0
I have no one to share my thoughts with	10	26	22	52	36	70	55	29	54	0
People expect too much from me	22	53	27	69	60	52	62	3	6	0
People are usually not interested in my achievements	11	42	29	68	83	62	38	5	16	0
I'm easily embarrassed	31	52	60	56	53	59	33	10	0	0
I feel as if many people do not understand me	23	43	50	83	63	49	37	2	4	0
I feel threatened	6	21	34	52	49	83	68	21	20	0
I often get nervous unnecessarily	49	76	63	63	47	32	14	6	4	0
I feel bad when I walk into a room full of people	28	42	44	49	55	56	44	30	6	0
I feel uncomfortable	27	29	41	69	65	69	40	10	4	0
People talk about me behind my back	18	26	27	74	54	65	64	12	14	0
I'm convinced that others are doing better than me	28	43	36	77	63	50	46	7	4	0
I feel guilty for bringing bad luck to others	6	0	11	31	23	67	65	79	72	0
It annoys me when I start to wonder about how other people feel about me	14	25	32	54	42	69	73	29	16	0
I'm not outgoing	14	20	35	54	64	76	50	27	14	0
I only speak up in disputes when I know I'm right	42	92	73	64	34	19	28	2	0	0
I still think about what others expect of me	27	58	41	64	54	48	51	7	4	0
Average number of points						Group I	66.6 ± 22.2 points			
						Group II	85.01 ± 28.3 points			
Minimum number of points						Group I	26			
						Group II	52			
Maximum number of points						Group I	121			
						Group II	117			

Table S4a. Attitudes of respondents towards older people according to the Kogan scale.

	**Average scores on the Kogan scale**		***p***
	**Young people (*n* = 177)**	**Seniors (*n* = 177)**	
**Negative attitude**			
It would probably be better if most old people lived in residential units with people their own age	3.6 ± 1.5	2.3 ± 1.3	<0.001
There is something different about most people; it's hard to find out what makes them tick	2.9 ± 1.4	3.9 ± 1.1	<0.001
Most old people get set in their ways and are unable to change	2.1 ± 1.3	4.2 ± 1.2	<0.001
Most old people would prefer to quit work as soon as pensions or their children can support them	4.3 ± 1.3	2.7 ± 1.4	<0.001
Most old people tend to let their homes become shabby and unattractive	4.5 ± 1.1	2.8 ± 1.3	<0.001
It's foolish to claim that wisdom comes with age	3.5 ± 1.7	3.4 ± 1.4	NS
Old people have too much power in business and politics	3.4 ± 1.5	3.1 ± 1.2	<0.05
Most old people make others feel ill at ease	4.4 ± 1.2	2.99 ± 1.3	<0.001
Most old people bore others by their insistence on talking "about the good old days"	4.9 ± 1.3	3.7 ± 1.3	<0.001
Most old people spend too much time prying into the affairs of others and giving unsought advice	2.9 ± 1.4	4.1 ± 1.2	<0.001
If old people expect to be liked, their first step should be to try to get rid of their irritating faults	4.3 ± 1.1	4.1 ± 1.1	0.095
In order to maintain a nice residential neighbourhood, it would be best if too many old people did not live in it	4.4 ± 1.4	2.95 ± 1.5	<0.001
There are a few exceptions, but in general, most old people are pretty much alike	3.8 ± 1.4	2.6 ± 1.3	<0.001
Most old people should be more concerned with their personal appearance; they're too untidy	3.98 ± 1.3	3.6 ± 1.5	0.01
Most old people are irritable, grouchy, and unpleasant	4.2 ± 1.3	2.6 ± 1.3	<0.001
Most old people are constantly complaining about the behaviour of the younger generation	2.8 ± 1.4	3.5 ± 1.4	<0.001
Most old people make excessive demands for love and reassurance more than anyone else	2.6 ± 1.1	4.4 ± 1.1	<0.001
**Positive attitudes**			
It would probably be better if most people lived in residential units with younger people	3.5 ± 1.4	3.4 ± 1.5	NS

Most old people are really no different from anybody else; they're as easy to understand as younger people	2.8 ± 1.4	3.5 ± 1.3	<0.001
Most old people are capable of new adjustments when the situation demands it	2.8 ± 1.4	3.6 ± 1.3	<0.001
Most old people would prefer to continue working just as long as they can instead of being dependent on anybody	4.3 ± 1.4	3.9 ± 1.5	<0.001
Most old people can generally be counted on to maintain a clean, attractive home	3.7 ± 1.3	3.6 ± 1.4	NS
People grow wiser with the coming of old age	3.5 ± 1.5	3.8 ± 1.4	NS
Old people should have power in business and politics	2.5 ± 1.1	2.8 ± 1.3	0.02
Most old people are very relaxing to be with	3.3 ± 1.3	3.1 ± 1.4	NS
One of the most interesting and entertaining qualities of most old people is their accounts of their past experiences	4.5 ± 1.1	4.1 ± 1.1	<0.001
Most old people tend to keep to themselves and give advice only when asked	2.6 ± 1.1	2.9 ± 1.2	0.022
When you think about it, old people have the same faults as everybody else	4.6 ± 1.3	4.3 ± 1.1	NS
You can count on finding a nice residential neighbourhood when there is a sizeable number of old people living in it	2.9 ± 1.3	2.7 ± 1.3	NS
It is evident that most old people are very different from one another	3.98 ± 1.4	4.4 ± 1.1	0.002
Most old people seem quite clean and neat in their personal appearance	3.6 ± 1.3	3.8 ± 1.4	NS
Most old people are cheerful, agreeable, and good-humoured	3.5 ± 1.3	2.3 ± 1.3	
I seldom hear old people complaining about the behaviour of the younger generation	2.3 ± 1.1	3.0 ± 1.4	<0.001
Most old people need no more love and reassurance than anyone else	2.3 ± 1.1	2.5 ± 1.3	NS
